# The importance of molecular diagnosis in severe sepsis determined by *Neisseria meningitidis* in the intensive care unit

**DOI:** 10.1186/1471-2334-13-S1-P38

**Published:** 2013-12-16

**Authors:** Mădălina Maria Merişescu, Monica Luminos, Gheorghiță Jugulete, Anca Drăgănescu, Angelica Vişan, Anuța Bilaşco, Camelia Kouris, Sabina Șchiopu, Dragoş Florea, Endis Osman, Adrian Streinu-Cercel

**Affiliations:** 1 National Institute for Infectious Diseases “Prof. Dr. Matei Balş”, Bucharest, Romania; 2 Carol Davila University of Medicine and Pharmacy, Bucharest, Romania

## Background

Infections caused by *Neisseria meningitidis* are an important cause of infant mortality worldwide. They take many clinical forms from simple respiratory infections to purpura fulminans, a severe form of sepsis, fatal in many cases. PLEX-ID is a novel method for etiologic diagnosis of bacterial infections. It can detect bacterial DNA found in various pathological products.

We studied the correlation of data obtained by classical culture methods versus molecular method results and the clinical and biological evolution of the patients under treatment.

## Methods

We conducted a 24 months study from January 2011 to December 2012 on children admitted in the Intensive Care Unit of the National Institute for Infectious Diseases “Prof. Dr. Matei Balş” for severe forms of meningococcal sepsis. Positive diagnosis of sepsis was established with classical methods, clinical and laboratory criteria (hemocultures, cerebrospinal fluid cultures), as well as PLEX-ID detection. The patients were divided into two groups based on the above mentioned criteria.

## Results

In the 24 months of study, 17 children were admitted to our clinic for meningococcal sepsis. In the first study group, we included 8 patients with severe meningococcal diseases. The diagnosis was established in these cases with classical methods. The mortality was 37.5% (3 of 8 cases). The second study group contained 9 patients diagnosed by molecular and conventional methods. In this group, 8 patients had favorable clinical evolution (88.8%).

## Conclusion

*Neisseria meningitidis* was found in the first 6 hours from admission and this lead to a decrease in mortality from 37.5% to 11.2%. The major advantage of this new method is the possibility to establish the etiological diagnosis in the early hours of patient admission.

